# Can Voice Pitch Be Preserved in Patients after Transoral Endoscopic Thyroidectomy Vestibular Approach?

**DOI:** 10.3390/jcm9092777

**Published:** 2020-08-27

**Authors:** Mi Ra Kim, Yeong Jun Park, Byung Whoo Park, Taekyung Suh, Sang-Yeon Kim, Tae Hoon Moon, Dong Hyun Lee, Jun-Ook Park

**Affiliations:** 1 Department of Otorhinolaryngology-Head and Neck Surgery, Haeundae Paik Hospital, Inje University College of Medicine, Busan 48108, Korea; enthns@naver.com (M.R.K.); gtgtyyu@gmail.com (Y.J.P.); H80484@paik.ac.kr (B.W.P.); dowithpride@naver.com (T.S.); tosiya@hanmail.net (S.-Y.K.); 2 Department of Otolaryngology-Head and Neck Surgery, Eunpyeong St. Mary’s Hospital, College of Medicine, The Catholic University of Korea, Seoul 03312, Korea; chosun_slp@naver.com (T.H.M.); cjidea@naver.com (D.H.L.)

**Keywords:** transoral thyroidectomy, functional voice outcomes, thyroid lobectomy, minimally invasive, endoscopic, transoral endoscopic thyroidectomy vestibular approach (TOETVA), voice quality

## Abstract

Introduction: Transoral endoscopic thyroidectomy vestibular approach (TOETVA) has become increasingly popular. Several reports have emphasized the safety and efficacy of this new approach. However, there is no report on functional voice outcomes, including voice pitch change after TOETVA. Methods: The functional voice outcomes of patients undergoing TOETVA were compared with those of patients undergoing conventional thyroidectomy. A total of 82 consecutive patients were included in the study: 44 underwent thyroid lobectomy via TOETVA (transoral group) and 38 underwent thyroid lobectomy via the classic cervical approach (open group). Thyroidectomy-related voice questionnaire (TVQ), perceptual voice analysis, fiberoptic laryngoscopic and videolaryngostroboscopic examinations, and acoustic analysis were carried out before and one month after surgery. The changes in these values after surgery and the differences between the transoral and open groups were analyzed. Results: We found no significant postoperative change in voice workups in either group. The mean high pitch decreased (from 367.91 ± 120.98 to 325.80 ± 100.86 Hz, *p* = 0.069) in the transoral group, but statistical significance was not attained. Clinically significant changes in pitch (postoperative change in speaking fundamental frequency, ΔSFF ≥ 12) after surgery were evident in seven (15.91%) patients in the transoral group and eight (21.05%) patients in the open group without significant difference (*p* = 0.579). Conclusions: This is the first study to assess functional voice outcomes (including pitch) after TOETVA compared with conventional open surgery. TOETVA was associated with good voice outcomes without any significant drop in pitch.

## 1. Introduction

Robotic and endoscopic thyroid surgeries have become increasingly popular. Over 20 remote approaches to the thyroid gland have been used to avoid visible neck scarring [1,2]. The recent transoral endoscopic thyroidectomy vestibular approach (TOETVA), in which the thyroid is safely resected via three intra-oral mucosal incisions (thus without any skin incision), is attractive [2,3,4,5]. Several reports have emphasized the safety and efficacy of this new approach [2,3,4,5]. The authors have performed TOETVA since 2016 and confirmed that this approach very safely preserves the recurrent laryngeal nerve, as reported in many other studies [2,3,5]. The principal cause of vocal dysfunction after thyroid surgery is iatrogenic injury to the recurrent laryngeal nerve. However, many patients suffer from minor voice problems after surgery, including frequent vocal fatigue and/or an inability to form high-pitched sounds and sing, even in the absence of recurrent laryngeal nerve injury and visible vocal cord paralysis [6,7,8,9]. Postoperative lowering of voice pitch is rather common (18% of patients) [10,11,12,13]. Several hypotheses have been proposed, such as inadvertent damage to the external branch of the superior laryngeal nerve (EBSLN) during surgery, postoperative adhesion of the strap muscle, changes in the laryngeal mucosa after thyroidectomy, and damage to the vocal cords caused by orotracheal intubation during surgery [8,9,10,11,13,14,15,16]. However, no explanation has been universally accepted.

Although the recurrent laryngeal nerves are safely preserved during TOETVA, no report on functional voice outcomes after TOETVA has appeared. How many patients experience lowered pitch after TOETVA? Are there any new surgery-related risk factors? Here, we investigated the functional voice outcomes of patients undergoing TOETVA compared with those of patients undergoing conventional thyroidectomy.

## 2. Methods

### 2.1. Study Design

We reviewed the medical records of 83 consecutive patients who underwent thyroid lobectomy via TOETVA or conventional open surgery from January 2018 to September 2019 in our hospitals. All were advised to undergo pre- and postoperative (at one month) voice workups. Those who underwent thyroid lobectomy were included. The exclusion criteria were (1)age < 20 or > 65 years, (2) total thyroidectomy for any reason,(3) combined with central or lateral compartment neck dissection, (4) thyroid cancer with any extrathyroidal extension, (5) history of surgical treatment or radiation to the head-and-neck and/or mediastinum, (6) any preoperative benign pathological lesion of the larynx (vocal polyps, vocal nodules, or vocal cord paralysis), (7) pre- or postoperative vocal cord paralysis, and (8) failure to complete voice workup. The Institutional Review Board of Haeundae Paik Hospital, Inje University, approved the study (IRB file no. 2017-12-011-002).

### 2.2. Thyroid Lobectomy

Patients were not randomly assigned to transoral or open thyroidectomy. Transoral endoscopic thyroidectomy was performed in patients who met the following inclusion criteria: (1) a request for a new surgical approach that avoids neck scarring, (2) thyroid cancer without any extrathyroidal extension or lymph node metastasis evident on preoperative ultrasonography, and (3) thyroid cancer < 2.5 cm in diameter or a benign tumor < 8 cm in diameter [5,17]. The detailed surgical procedure was described in our previous paper [5,17]. All surgeries were performed in the same manner by a single surgeon (J-O Park).

### 2.3. Functional Voice Analysis: Subjective

#### 2.3.1. Thyroidectomy-Related Voice Questionnaire (TVQ)

The TVQ is a self-assessment tool that measures voice quality after thyroidectomy; the TVQ was developed and validated in our institution [18,19,20]. It consists of 20 questions exploring voice symptoms (*n* = 10) and swallowing and laryngopharyngeal reflux (*n* = 10). Each question is scored from 0 (no symptoms) to 4 (maximum symptoms), and the scores are summed. The total TVQ score thus ranges from 0 (no symptoms) to 80 (maximum voice and swallowing symptoms). All patients were asked to complete the TVQ before and one month after surgery.

#### 2.3.2. Perceptual Voice Analysis

The grade, roughness, breathiness, asthenia, and strain (GRBAS) score is a widely accepted objective measure of voice. Grade (G) is the overall extent of deviance, roughness (R) is irregular fluctuation of the fundamental frequency [F0], breathiness (B) is a turbulent noise produced by air leakage, asthenia (A) is overall voice weakness, and strain (S) is an impression of tenseness or excess effort. All were categorized as 0 (normal), 1 (slight disturbance), 2 (moderate disturbance), or 3 (severe disturbance). Voice samples were recorded as patients read “Sanchaek (a walk)” (a Korean text) at a comfortable volume and rate. The GRBAS was scored at the end of the evaluation. Next, the recorded voices were replayed and the scores revised. Scoring was performed by two speech therapists and two otolaryngologists, who were blinded to which surgery the patients received, working in consensus.

### 2.4. Functional Voice Analysis: Objective

#### 2.4.1. Fiberoptic Laryngoscopic and Videolaryngostroboscopic Examinations

Fiberoptic laryngoscopy (Machida Instruments, Tokyo, Japan) and videolaryngostroboscopy (model 9200C; KayPENTAX, Lincoln Park, NJ, USA) were used to evaluate the vocal folds. Fiberoptic laryngoscopic and videolaryngostroboscopic findings were reviewed by two otolaryngologists, who had no patient information, working in consensus.

#### 2.4.2. Acoustic Analysis

Acoustic analysis is a validated tool employed to quantitatively characterize voice in terms of dysphonia. Patients were instructed to vocalize the vowel “a” at a comfortable volume and constant pitch. Each pronunciation was recorded at a constant mouth-to-microphone distance of 5 cm using Computerized Speech Lab (model 4150; KayPENTAX). All recordings were made in a quiet room. Each patient sustained the “a” sound for at least 3 s at a comfortable pitch. The task was repeated at least four times, and the fourth trial usually employed the Multi-Dimensional Voice Program (model 5105, ver. 3.1.7; KayPENTAX). The parameters considered were the fundamental frequency (F0, Hz), perturbations of the fundamental frequency (jitter, %), amplitude (shimmer, %), glottal noise (i.e., the noise-to-harmonic ratio), speaking fundamental frequency (SFF, Hz), pitch range (Hz), high pitch (Hz), and low pitch (Hz). The SFF is the average fundamental frequency (the lowest frequency of a complex periodic sound) measured during performance of a vocal or speech task, and it is a basic acoustic measure used for clinical evaluation of voice disorders, such as a lowered pitch. To identify patients with lower-pitched voices, SFFs were compared before and after surgery. Changes in all patients were calculated (postoperative change in SFF, ΔSFF = preoperative value of SFF – postoperative value of SFF, Hz). If the ΔSFF was > 12 Hz, the patient was considered to have a lower-pitched voice [12,13,21]. The software defines jitter values up to N < 1.1% and shimmer values up to N < 3.8% as normal. The normal noise-to-harmonic ratio is N < 0.2. The results of the acoustic analysis were judged by two otolaryngologists who were blinded to which surgery the patients received and who reached consensus.

### 2.5. Statistical Analyses

Statistical analyses were performed using IBM SPSS Statistics software (ver. 25.0) (SPSS Inc., Chicago, IL, USA). To determine whether our sample size had sufficient statistical power, we performed an a priori power analysis using the two-sided hypothesis test at an alpha level of 0.05 and a statistical power of 80%. Sixty-eight patients were required. To allow for exclusion, 83 patients were included in the study. The demographic, clinical, perceptual voice analysis, videolaryngostroboscopic, and objective acoustic voice analysis data were compared between the TOETVA and open thyroidectomy groups using the *t*-test, Mann–Whitney test, Wilcoxon’s test, and Fisher’s exact test, as appropriate. All data are presented as means ± standard deviation. A *p*-value < 0.05 was taken to indicate statistical significance.

## 3. Results

Among the 83 patients, one patient in the TOETVA group who showed postoperative transient vocal cord paralysis (and recovered at postoperative one month follow up) in the videolaryngostroboscopic evaluation was excluded from the study. A total of 82 consecutive patients were included in the study: 44 underwent thyroid lobectomy via TOETVA (transoral group) and 38 underwent thyroid lobectomy via the classic cervical approach (open group). The patient characteristics and preoperative results of the subjective and objective voice analyses are summarized in Table 1. The mean patient age was lower in the transoral than open group (42.8 ± 12.6 years vs. 52.0 ± 12.6 years, *p =* 0.003*). No other patient characteristic differed significantly between the two groups. The preoperative auditory perceptual evaluations, total TVQ questionnaire scores, and acoustic voice analysis data did not differ significantly between the groups.

**Figure 1:**
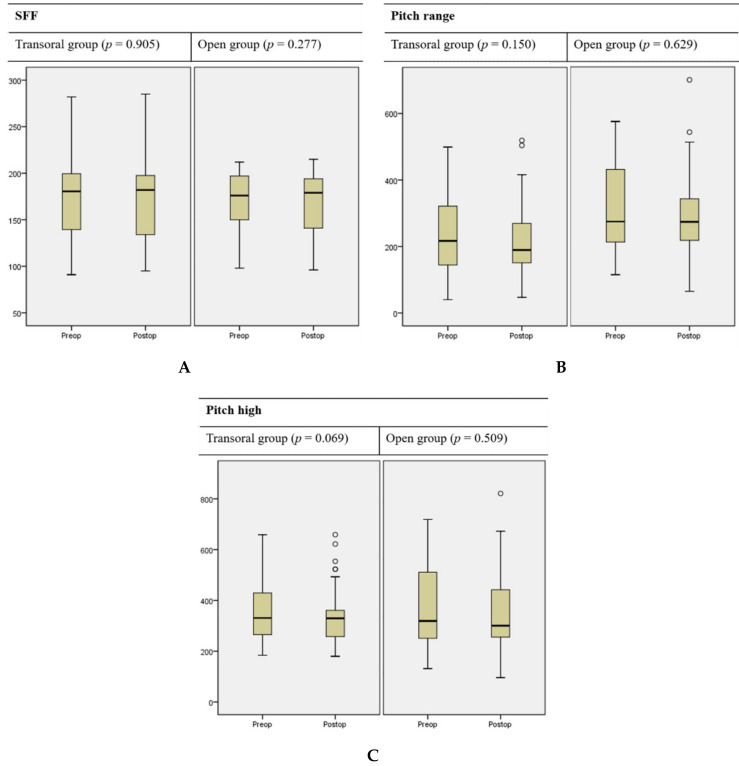
Postoperative changes in (**A**) the speech fundamental frequency (SFF), (**B**) the pitch range, and (**C**) high pitch in patients who underwent transoral endoscopic thyroidectomy vestibular approach (transoral group) or open thyroidectomy (open group). Circles denote strong outliers.

**Figure 2:**
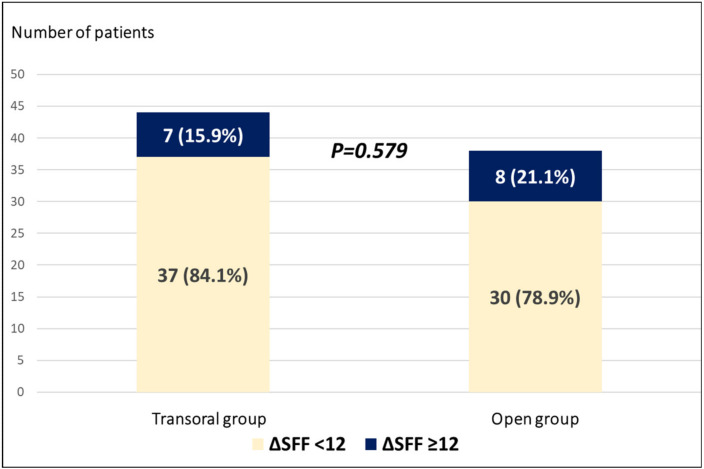
The proportions of patients who exhibited clinical changes in the SFF (ΔSFF ≥ 12) after transoral endoscopic thyroidectomy vestibular approach (TOETVA) or open thyroidectomy. Abbreviations: SFF, speech fundamental frequency; difference (Δ), preoperative value − postoperative value; TOETVA, transoral endoscopic thyroidectomy vestibular approach; Open, open thyroidectomy.

The postoperative changes in subjective voice parameters are summarized in Table 2. We found no significant change in the GRBAS score in either group after surgery. The TVQ score increased in both groups, but statistical significance was not attained. The postoperative changes in pitch parameters are summarized in Table 3 and Figure 1. We found no significant postoperative change in F0, SFF, pitch range, or high pitch in either group. The mean high pitch decreased (from 367.91 ± 120.98 to 325.80 ± 100.86 Hz, *p* = 0.069) in the transoral group, but statistical significance was not attained. Clinically significant changes in pitch (ΔSFF ≥ 12) after surgery were evident in seven (15.91%) patients in the transoral group and eight (21.05%) patients in the open group; no significant between-group difference was apparent *(p =* 0.579) (Figure 2).

## 4. Discussion

The potential causes of lowered pitch after thyroid surgery include EBSLN injury, laryngotracheal fixation, impaired vertical movement, temporary dysfunction of the cricothyroid muscle, strap muscle adhesion, modification of the laryngeal blood supply, laryngeal injury associated with endotracheal intubation, and psychological problems [13,22,23]. The EBSLN is often injured during dissection of the superior pole of the thyroid gland, rendering the cricothyroid muscle dysfunctional. The SFF is lowered and voice performance deteriorates in terms of the production of high-frequency sounds, which can be serious if patients are singers or actors.

**Figure 3:**
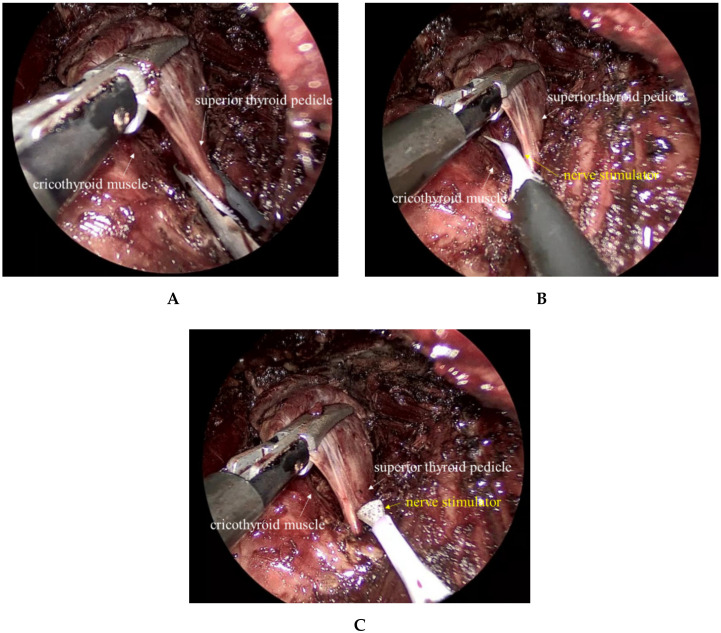
Right superior pole dissection during the transoral endoscopic thyroidectomy vestibular approach (TOETVA). (**A**) The external branch of the superior laryngeal nerve (EBSLN) can be damaged by the energy device during ligation of the superior thyroid vessels. The use of intraoperative neural monitoring during TOETVA: (**B**) a nerve stimulator is placed between the superior thyroid pedicle and the cricothyroid muscle (CTM) to stimulate the EBSLN, causing the CTM to visibly twitch (true positive stimulation). (**C**) The stimulator is then applied to the superior thyroid pedicle (negative stimulation of the EBSLN) (true negative stimulation).

During TOETVA, the surgeon views the thyroid gland from the cranial to caudal direction; the superior poles are poorly visible, but the lower poles are obvious. For an inexperienced surgeon, superior pole dissection is thus the most difficult part of the procedure. During TOETVA, after an avascular space between the trachea and thyroid has been established, the space is widened and opened to allow the thyroid to be grasped using a grasper (with one blade in the avascular space and the other outside of the thyroid) and pulled inferomedially to expose the superior pole. After the end of the superior pole has been sufficiently exposed and the superior thyroid vessels identified, an energy device is used to ligate the vessels. During these steps, the EBSLN could be bitten by the grasper or damaged during energy ligation of the superior thyroid vessels (Figure 3A). Therefore, we hypothesized that TOETVA is associated with a risk of EBSLN injury during superior pole dissection, causing a significant drop in pitch. We measured various pitch-related parameters including the F0, SFF, pitch range, low pitch, and high pitch before and one month after surgery. The SFF, F0, and pitch range of patients who underwent TOETVA did not decrease. High pitch tended to decrease in the transoral group, but statistical significance was not attained. Approximately 15% of patients exhibited clinically significant lower-pitched voices (ΔSFF > 12 Hz) one month after TOETVA, similar to the proportion exhibiting lower-pitched voices after open thyroidectomy (seen in this study and our previous reports) [12,13]. Our results suggest that TOETVA does not impose any additional pitch risk to those imposed by conventional open surgery. However, our work had certain limitations. We did not use laryngeal electromyography to evaluate damage to the EBSLN. In addition, the follow-up duration was too short to adequately determine the time course of voice recovery after surgery. Although significant deteriorations in voice quality usually develop immediately after thyroidectomy [10,13,24], we did not explore when the voice outcomes were poorest, when recovery commenced, or when the parameters returned to pre-surgery levels. Further studies are needed.

We have recently included intraoperative neural monitoring (IONM) to ensure non-entrapment of the EBSLN during superior pole dissection; we adhere to the standard of an international neural monitoring study group [25]. As superior pole dissection commences, we usually toggle a nerve stimulator between the superior thyroid pedicle and the cricothyroid muscle. We first positively stimulate the EBSLN, and the muscle visibly twitches (true positive stimulation in Figure 3B). We next stimulate the superior thyroid pedicle that is to be divided (negative stimulation of the EBSLN) (true negative stimulation in Figure 3C). We will later report whether IONM usefully and reliably preserves the EBSLN during TOETVA.

## 5. Conclusions

This is the first study to assess functional voice outcomes (including pitch) after TOETVA compared with conventional open surgery. TOETVA was associated with good voice outcomes without any significant drop in pitch. However, a further study featuring IONM and laryngeal electromyography is needed to confirm the safety of TOETVA in terms of EBSLN preservation.

## Figures and Tables

**Table 1:** Clinical characteristics of the patients in both groups.

Characteristics	Transoral Group (*n* = 44)	Open Group (*n* = 38)	*p*-Value
Age (years)	42.8 ± 12.6	52.0 ± 12.6	0.003 *
Gender			0.516
Male	12	8	
Female	32	30	
Pathology of tumor			0.848
Benign	4	3	
Malignant	40	35	
Size of tumor	1.19 ± 1.06	1.22 ± 1.48	0.569
Number of tumors	1.25 ± 0.72	1.50 ± 1.06	0.229
Current smoking			0.828
Yes	4	4	
No	40	34	
Alcohol consumption			0.383
Yes	13	8	
No	31	30	
Type of voice user			0.075
Professional voice user	20	10	
Nonvocal professionals	24	28	
Preoperative voice workup			
Perceptual evaluation			
Grade	0.14 ± 0.41	0.05 ± 0.23	0.318
Roughness	0.07 ± 0.33	0.00 ± 0.00	0.186
Breathiness	0.09 ± 0.29	0.00 ± 0.00	0.058
Asthenia	0.00 ± 0.00	0.05 ± 0.23	0.126
Strain	0.00 ± 0.00	0.00 ± 0.00	1.000
Subjective analysis			
TVQ scores	14.41 ± 13.79	15.71 ± 17.20	0.716
Acoustic voice analysis			
F0 (Hz)	187.45 ± 59.29	179.98 ± 42.67	0.463
SFF (Hz)	172.15 ± 44.03	170.20 ± 32.36	0.625
Jitter (%)	1.11 ± 1.09	0.89 ± 0.64	0.874
Shimmer (%)	3.52 ± 1.80	3.43 ± 1.40	0.915
NHR	0.13 ± 0.04	0.13 ± 0.04	0.679
Pitch range (Hz)	249.49 ± 123.21	260.77 ± 93.17	0.444
Pitch low (Hz)	118.42 ± 36.98	110.98 ± 26.57	0.437
Pitch High (Hz)	367.91 ± 120.98	372.92 ± 101.04	0.539

Abbreviations: *n*, number of patients; *, statistical significance; TOETVA, transoral endoscopic thyroidectomy vestibular approach; TVQ, thyroidectomy-related voice questionnaire; F0, fundamental frequency; SFF, speech fundamental frequency; NHR, noise to harmonic ratio; SD, standard deviation.

**Table 2:** Subjective voice outcomes after use of the transoral endoscopic thyroidectomy vestibular approach (TOETVA) or open thyroidectomy.

	Transoral Group (*n* = 44)			Open Group (*n* = 38)		
	Preoperative	Postoperative	*p*-Value	Preoperative	Postoperative	*p*-Value
Perceptual evaluation						
Grade	0.14 ± 0.41	0.16 ± 0.37	0.705	0.05 ± 0.23	0.05 ± 0.23	1.000
Roughness	0.07 ± 0.33	0.05 ± 0.21	0.655	0.00 ± 0.00	0.03 ± 0.16	0.317
Breathiness	0.09 ± 0.29	0.11 ± 0.32	0.564	0.00 ± 0.00	0.03 ± 0.16	0.317
Asthenia	0.00 ± 0.00	0.00 ± 0.00	1.000	0.05 ± 0.23	0.03 ± 0.16	0.564
Strain	0.00 ± 0.00	0.02 ± 0.15	0.317	0.00 ± 0.00	0.00 ± 0.009	1.000
TVQ scores	14.41 ± 13.79	20.10 ± 9.31	0.097	15.71 ± 17.20	27.66 ± 20.67	0.078

Abbreviations: *n*, number of patients; TOETVA, transoral endoscopic thyroidectomy vestibular approach; TVQ, thyroidectomy-related voice questionnaire.

**Table 3:** Voice pitch changes after use of the transoral endoscopic thyroidectomy vestibular approach (TOETVA) or open thyroidectomy.

	Transoral Group (*n* = 44)				Open Group (*n* = 38)			
Parameters	Preoperative	Postoperative	Difference (Δ)	*p*-Value	Preoperative	Postoperative	Difference (Δ)	*p*-Value
F0 (Hz)	187.45 ± 59.29	187.36 ± 57.30	0.09 ± 21.40	0.986	179.98 ± 42.67	179.38 ± 42.22	0.60 ± 18.20	0.658
SFF (Hz)	172.15 ± 44.03	170.85 ± 44.00	1.30 ± 10.58	0.905	170.20 ± 32.36	168.16 ± 32.95	2.04 ± 12.09	0.277
Pitch range (Hz)	249.49 ± 123.21	218.38 ± 106.97	31.11 ± 108.43	0.150	260.77 ± 93.17	251.51 ± 74.92	9.27 ± 86.03	0.629
Pitch low (Hz)	118.42 ± 36.98	117.41 ± 41.81	1.01 ± 33.69	0.665	110.98 ± 26.57	110.33 ± 25.10	0.65 ± 26.43	0.829
Pitch High (Hz)	367.91 ± 120.98	325.80 ± 100.86	32.11 ± 99.17	0.069	372.92 ± 101.04	361.48 ± 81.93	11.44 ± 84.75	0.509
Jitter (%)	1.11 ± 1.09	1.01 ± 1.09	N/A	0.243	0.89 ± 0.64	0.92 ± 0.77	N/A	0.557
Shimmer (%)	3.52 ± 1.80	3.28 ± 1.76	N/A	0.294	3.43 ± 1.40	3.53 ± 1.46	N/A	0.902
NHR	0.13 ± 0.04	0.13 ± 0.03	N/A	0.595	0.13 ± 0.04	0.13 ± 0.02	N/A	0.220

Abbreviations: *n*, number of patients; TOETVA, transoral endoscopic thyroidectomy vestibular approach; F0, fundamental frequency; SFF, speech fundamental frequency; NHR, noise-to-harmonic ratio; difference (Δ), preoperative value − postoperative value; N/A, not applicable.
